# Complete mitochondrial genome of the Korean endemic firefly, *Luciola unmunsana* (Coleoptera: Lampyridae)

**DOI:** 10.1080/23802359.2020.1806753

**Published:** 2020-08-14

**Authors:** Min Jee Kim, Jeong Sun Park, Iksoo Kim

**Affiliations:** a Department of Applied Biology, College of Agriculture & Life Sciences, Chonnam National University, Gwangju, Republic of Korea; b Herbal Medicine Resources Research Center, Korea Institute of Oriental Medicine, Naju, Republic of Korea

**Keywords:** Luciola unmunsana, mitochondrial genome, firefly, endemic species, phylogeny

## Abstract

In this study, we announce the complete mitochondrial genome (mitogenome) of the Korean endemic firefly, *Luciola unmunsana* Doi, 1931. The full-length circular genome was 15,858 bp, with 77.94% A/T content. It contained the typical set of 37 metazoan genes: 13 protein-coding genes (PCGs), 22 transfer RNA (tRNA) genes, and 2 ribosomal RNA (rRNA) genes, as well as an A + T-rich region. The gene arrangement of the species is identical to that of the ancestral arrangement found in the majority of insects. The maximum-likelihood tree, built using all PCGs and two rRNAs via randomized accelerated maximum likelihood (RAxML) showed that *L. unmunsana* was grouped as a sister to *L. curtithorax* with the highest nodal support. However, another *Luciola* species clustered with the *Aquatica* species such that the genus *Luciola* was a non-monophyletic group. Therefore, more sampling is required to clarify the phylogeny of *Luciola*.

The genus *Luciola* Laporte, 1833 is a firefly (subfamily Luciolinae) (Stanger-Hall et al. 2007), and three species are found in Korea: *L. lateralis*, *L. unmunsana*, and *L. papariensis* (Kang 2012). Among them, *L. unmunsana* Doi 1931 (Coleoptera: Lampyridae), is an endemic species in Korea (Doi 1931). *Luciola unmunsana* and *L. papariensis* are nearly identical morphologically. A single morphological character, which can distinguish the two species, is the color pattern of the pronotum (Kang 2012). Recently, Han et al. (2020) studied the phylogeny of the *Luciola* species to elucidate the species status of the two Korean *Luciola* species using the DNA barcode region. However, their results showed that the lineages of the two species split into several groups, although intergroup relationship was not distinct enough to consider each an independent species. In this study, we sequenced the complete mitochondrial genome (mitogenome) of *L. unmunsana* (GenBank accession number: MT134039) for subsequent mitogenome-based phylogenetic analysis.

An *L. unmunsana* adult was collected from Mt. Unmunsan, Cheongdo, Gyeongsangbuk-do Province in Korea (35°39′17.7″N, 128°57′57.4″E) and total DNA was extracted from two hind legs. The leftover DNA and the specimen were deposited at the Chonnam National University, Gwangju, Korea, under the accession number CNU12790. Full-length mitogenome sequence data for *L. unmunsana* were obtained via next-generation sequencing using the MGISEQ-2000 sequencing platform (MGI Tech Co. Ltd, Shenzhen, China). Genome construction was performed using *de novo* assembly. Owing to the precise nature of final genome sequence, no additional Sanger-based sequencing was conducted. Phylogenetic analysis was performed within the scope of Luciolinae with 15 available mitogenomes, including *L. unmunsana,* using randomized axelerated maximum likelihood (RAxML) (Stamatakis 2014). For the analysis, 13 protein-coding genes (PCGs) and 2 ribosomal RNA (rRNA) genes were aligned and a total length of 12,517 bp (excluding gaps) was analyzed using the substitution model, GTR + Gamma + I.

We assembled the 15,858-bp long complete mitogenome of *L. unmunsana* from 137,521,575 high-quality clean reads. The genome contained 13 PCGs, 22 transfer RNAs (tRNAs), 2 rRNAs, and 1 major non-coding A + T-rich region that was 1247 bp long. The overall A/T nucleotide composition of the *L. unmunsana* mitogenome was as follows: 75.82% in the 13 PCGs, 77.94% in the whole genome, 79.57% in tRNAs, 80.72% in srRNA, 82.68% in lrRNA, and 88.13% in the A + T-rich region. The gene arrangement of *L. unmunsana* was identical to that of the ancestral arrangement found in the majority of insects (Boore 1999). The *L. unmunsana* mitogenome had the shortest length (16,385 bp on average) among mitogenomes of 16 Luciolinae members, which ranged from 15,967 bp (*Asymmetricata circumdata*; Luan and Fu 2016) to 16,882 bp (*L. curtithorax*; Hu and Fu 2018a).

*Luciola* species comprises non-monophyletic groups, placing the current *L. unmunsana* as a sister to *L. curtithorax* with the highest nodal support, whereas other species of *Luciola* were grouped together with species of *Aquatica* (Figure 1). Following Ballantyne and Lambkin’s (2009) study, wherein the genera are presented as polyphyletic groups using 343 morphological characters, a new genus, *Aquatica*, was proposed for some species of *Luciola* using behavioral and morphological evidence (Fu et al. 2010). Nevertheless, additional phylogenetic revision with the inclusion of extended taxa is required to further clarify the phylogeny of *Luciola*.

**Figure 1.**
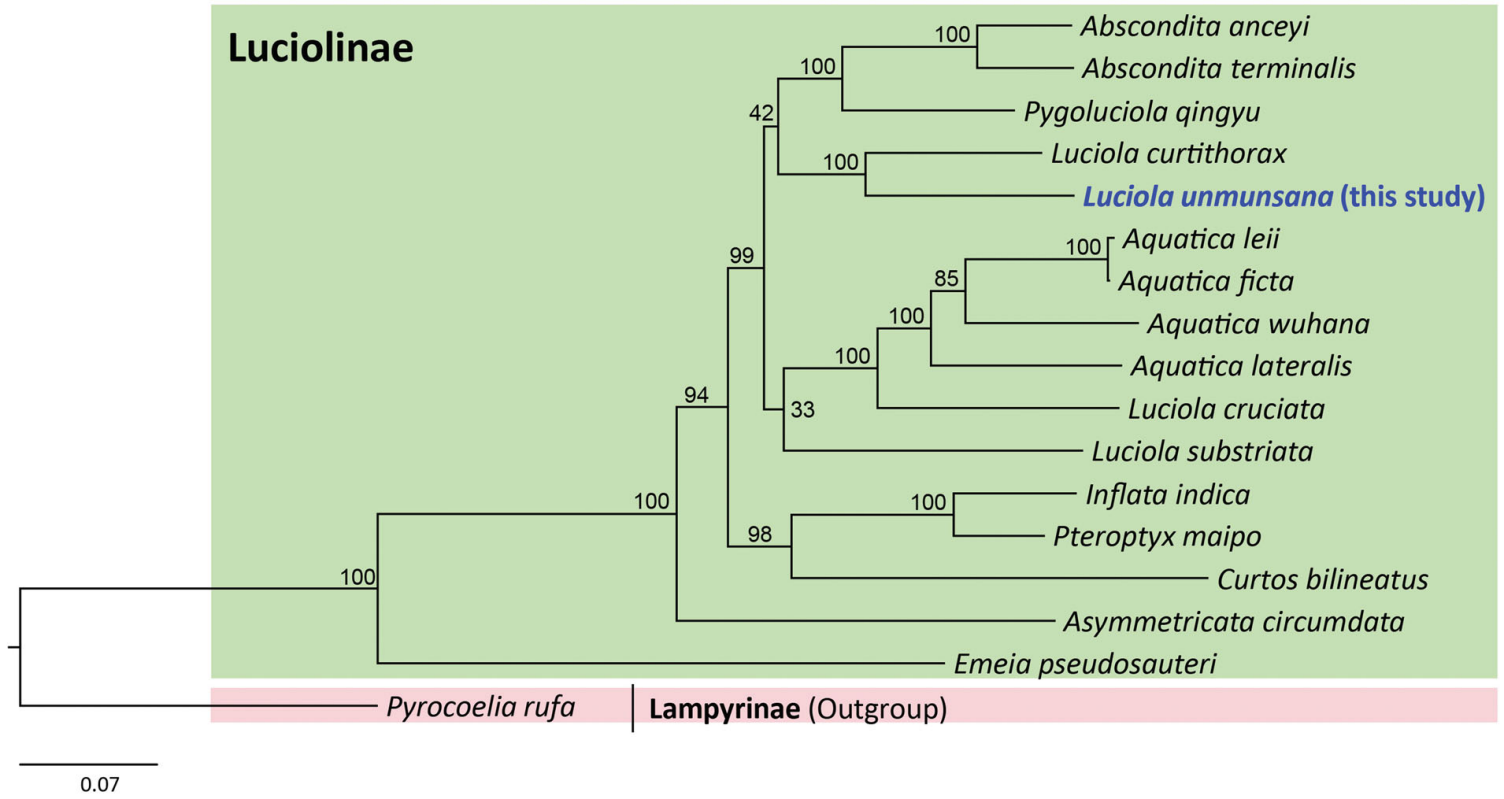
Phylogenetic tree for the subfamily Luciolinae. The maximum likelihood (ML) method was applied using randomized accelerated maximum likelihood (RAxML) ver. 8.0.24 (Stamatakis 2014), which was incorporated into the cyberinfrastructure for phylogenetic research (CIPRES) Portal ver. 3.1 (Miller et al. 2010). A six optimal partitioning scheme and substitution model (GTR + Gamma + I) were determined using PartitionFinder 2 with the Greedy algorithm (Lanfear et al. 2012, 2014, 2016). Phylogenetic trees were visualized using FigTree ver. 1.42 (http://tree.bio.ed.ac.uk/software/figtree/). The numbers at each node represent bootstrap percentages of 1,000 pseudoreplicates by ML analysis. The scale bar indicates the number of substitutions per site. Lampyrinae (*Pyrocoelia rufa*, MH352481; Bae et al. 2004) is used as an outgroup. GenBank accession numbers are as follows: *Abscondita anceyi*, MH020192 (Hu and Fu 2018b); *Abscondita terminalis*, MK292092 (Chen et al. 2019); *Pygoluciola qingyu*, MK292093 (Chen et al. 2019); *Aquatica leii*, KF667531 (Jiao et al. 2015); *Aquatica ficta*, KX758085 (Wang et al. 2017); *Aquatica wuhana*, KX758086 (Wang et al. 2017); *Luciola cruciata*, AB849456 (Maeda et al. 2017a); *Aquatica lateralis*, LC306678 (Maeda et al. 2017b); *Luciola curtithorax*, MG770613 (Hu and Fu 2018a); *Luciola substriata*, KP313820 (Mu et al. 2016); *Inflata indica*, MH427718 (Sriboonlert and Wonnapinij 2019); *Pteroptyx maipo*, MF686051 (Fan and Fu 2017); *Curtos bilineatus*, MK292114 (Chen et al. 2019); *Asymmetricata circumdata*, KX229747 (Luan and Fu 2016); and *Emeia pseudosauteri*, MK292112 (Chen et al. 2019).

## Data Availability

The data that support the findings of this study are openly available in Mendeley Data at http://dx.doi.org/10.17632/rh3cd4ztx7.1
